# Advances and prospects of precision nanomedicine in personalized tumor theranostics

**DOI:** 10.3389/fcell.2024.1514399

**Published:** 2024-12-05

**Authors:** Yuhang Mao, Juanping Xie, Fang Yang, Yan Luo, Juan Du, Hong Xiang

**Affiliations:** ^1^ School of Medicine, Ankang University, Ankang, China; ^2^ Ultrasound Medicine Department, Ankang Traditional Chinese Medicine Hospital, Ankang, China; ^3^ Shanxi Province Engineering and Technology Research Center for Development and Utilization of Qinba Traditional Chinese Medicine Resources, Ankang University, Ankang, China; ^4^ School of Modern Agriculture and Biotechnology, Ankang University, Ankang, China; ^5^ Department of Stomatology, Hengqin Hospital, First Affiliated Hospital of Guangzhou Medical University, Guangzhou, China

**Keywords:** tumor, personalized medicine, nanomedicine, treatment, diagnosis

## Abstract

Tumor, as the second leading cause of death globally, following closely behind cardiovascular diseases, remains a significant health challenge worldwide. Despite the existence of various cancer treatment methods, their efficacy is still suboptimal, necessitating the development of safer and more efficient treatment strategies. Additionally, the advancement of personalized therapy offers further possibilities in cancer treatment. Nanomedicine, as a promising interdisciplinary field, has shown tremendous potential and prospects in the diagnosis and treatment of cancer. As an emerging approach in oncology, the application of nanomedicine in personalized cancer therapy primarily focuses on targeted drug delivery systems such as passive targeting drug delivery, active targeting drug delivery, and environmentally responsive targeting drug delivery, as well as imaging diagnostics such as tumor biomarker detection, tumor cell detection, and *in vivo* imaging. However, it still faces challenges regarding safety, biocompatibility, and other issues. This review aims to explore the advances in the use of nanomaterials in the field of personalized cancer diagnosis and treatment and to investigate the prospects and challenges of developing personalized therapies in cancer care, providing direction for the clinical translation and application.

## 1 Introduction

Tumor, or cancer, is a disease characterized by abnormal proliferation and growth of cells, which can occur and proliferate everywhere to form a mass (Chen et al., 2023a; Wei et al., 2024). These malignant cells can invade surrounding tissues and even enter the bloodstream and lymphatic system, spreading to other organs of the body through metastasis (Kloosterman et al., 2024; Yang et al., 2024). Currently, cancer continues to pose a significant global health challenge, ranking as the second leading cause of death worldwide, following by the heart disease. And the lung carcinoma contributed to the highest mortality rate (Meyer et al., 2024). The tumorigenesis may be related to various factors such as genetics, environments and lifestyles. And the treatments of tumors typically include surgical resection, radiation therapy, chemotherapy, immunotherapy and targeted therapy (Chen et al., 2022a; Leggett et al., 2022). Although significant discoveries and achievements have been made in tumor treatment, the overall mortality rate related to cancer has remained relatively stable (Chen et al., 2022b). Every treatment has its drawbacks, including poor drug solubility, short half-life time, multidrug resistance, systemic and local non-targeted side effects (Boshuizen and Peeper, 2020; Kaur et al., 2023; Gao et al., 2021). Therefore, there’s an urgent need for continuous development of safer and more efficient treatment strategies. Additionally, improving early tumor diagnosis and imaging techniques can help detect and monitor tumor progression earlier, enabling a better understanding of the tumor progression and guiding treatment accordingly.

Personalized medicine, also known as precision medicine, relies on comprehensive analysis of patients’ genetic information, tumor characteristics, lifestyles and environmental factors to provide more precise and effective treatment strategies (Arrieta et al., 2023; Zhou et al., 2024a). Through comprehensive information gathering and analysis, clinicians can better understand the disease status of cancer patients, predict disease progression, and select the most appropriate treatment plans to improve treatment efficacy, reduce adverse reactions, mitigate drug resistance, enhance patient survival rates and improve quality of life. This has also spurred the development of novel treatment strategies, bringing forth more possibilities for cancer treatment (Ding et al., 2021; Selvakumar et al., 2022; Hua et al., 2021; Garcia et al., 2023). So, the personalized medicine is an indispensable approach and trend in cancer treatment.

Nanomedicine, as a promising interdisciplinary field, combines nanoscience with medicine, aiming to utilize nanotechnology for the disease diagnosis, treatment and prevention (Sindhwani et al., 2020). Nowadays, research in nanomedicine mainly focused on the design and fabrication of nanoscale materials, devices or systems, including nanodrug delivery systems, nano-diagnostic technologies, tissue engineering and regenerative medicine, and disease treatment using nanoparticles (Bhatia et al., 2022). Nanomedicine has shown tremendous potential and prospects in the diagnosis and treatment of tumors (Björnmalm et al., 2017). Certain nanomaterials can release encapsulated or conjugated bioactive substances based on the unique chemical and biological conditions of the tumor microenvironment, which enable effective drug accumulation at the tumor site, promotes uptake by tumor cells, so as to enhance the bioavailability of drugs (Zhou et al., 2023). Additionally, combining nanomaterials with therapeutic drugs and tumor imaging techniques can enhance anti-tumor effects while effectively monitoring tumor occurrence and development (Zhou et al., 2022; Liu et al., 2023). However, nanomedicine still faces some challenges, such as the safety and biocompatibility of nanomaterials, production costs and large-scale manufacturing. Nevertheless, with advancing technologies and continued research investments, nanomedicine holds the promise of bringing more innovations and breakthroughs to medicine in the future.

Ultimately, this review summarizes the latest advancements of nanomaterials in the field of personalized medicine for tumors, organizes the types and methods of nanomaterials used so far, mainly introduces the classification of nanomaterials, nanomaterial delivery systems, and their applications in tumor diagnosis and treatment, and further discusses their prospects and existing issues, aiming to provide direction for the clinical translation and application of nanomaterials in cancer personalized medicine.

## 2 Overview of nanomaterials and personalized treatment for tumor

Nanomaterials, with diameters ranging from just 1–100 nm, exhibit unique properties such as conductivity, stability, optical characteristics, and surface modification potential, giving them broad application prospects across multiple fields, including medicine, pharmacy, electronics, protein diagnostics, and cellular engineering. In medicine and biology, nanoparticles can serve as drug delivery carriers to enhance bioavailability, improve targeting, and reduce side effects. They have also achieved significant progress in diagnostic imaging and immunotherapy. In cancer treatment, nanoparticles can deliver drugs, genes, or immune modulators directly to tumor sites, allowing for localized therapeutic effects, enhanced efficacy, and reduced damage to healthy tissues. This targeted delivery capability makes nanoparticles a crucial tool in personalized cancer therapy. Additionally, certain nanomaterials show potential for use in photothermal and magnetic hyperthermia therapies, which have proven effective in localized heating to ablate tumors. Currently, with continuous innovations in nanomaterials, nanomedicine is making further strides in personalized cancer therapy and is gradually advancing towards integrated diagnostic and therapeutic approaches. In the future, nanomaterials are expected to play a more prominent role in early tumor diagnosis, real-time monitoring, and combined therapies, further enhancing the effectiveness of personalized treatments and offering patients safer, more efficient therapeutic options.

### 2.1 Development of nanomaterials

Three stages comprise the development of nanomedicine: the first took place between 1964, when the structure of liposomes was discovered, and 1995, when the US Food and Drug Administration (FDA) approved liposomal doxorubicin (DOX), also marketed as Doxil^®^ (Viana et al., 2021). The clinical validation and commercialization of nanomedicine constituted the second phase, which ran from 1995 to 2007 (Chen et al., 2022c). The third stage, from 2007 to the present, represented a period of rapid advancement in nanomedicine, marked by continuous emergence of various innovative technologies. Nanoparticles (NPs) are microscopic particles with sizes ranging from 1 to 100 nm. Due to their unique physical properties such as conductivity, stability, and optical characteristics, nanoparticles have become ideal subjects for research in biology and materials science (Shi et al., 2017; Aghebati-Maleki et al., 2020). Nanoparticles have been proven to have promising applications in various fields including medicine, pharmacy, tissue engineering, environment, energy, electronics, biomolecular and protein diagnostics, and cellular engineering (Hou and Liu, 2023). They are categorized based on their properties, shapes, and sizes into different classes, including carbon-based NPs, metal NPs, ceramic NPs, polymer NPs, lipid-based NPs, and virus-based NPs (Sarma et al., 2021; Sun et al., 2023). Polymer NPs, lipid NPs, and virus NPs belong to organic NPs (Bodunde et al., 2021; Vargas-Nadal et al., 2022), while carbon-based NPs, metal NPs, ceramic NPs, and semiconductor NPs belong to inorganic NPs (Bodunde et al., 2021; Li et al., 2019; Zhou et al., 2017; Greene et al., 2021), as detailed in Table 1.

**TABLE 1:** Classification of nanoparticles and their applications in tumors.

Classification	Type	Compositions	Applications	References
Organic NPs	Lipid NPs	Liposomes, Solid Lipid Nanoparticles (SLN), and Nanostructured Lipid Carriers (NLC)	Drug delivery, drug carriers and RNA release	Hao et al. (2020)
	Virus NPs	Genetic Engineering VNPs and Chemical Engineering VNPs	Genetic Engineering VNPs are used in vaccine development; Chemical Engineering VNPs are often utilized in targeted drug therapy for tumors and biomedical imaging	Zhdanov (2021) and Chung et al. (2020)
	Polymer NPs	Organic Nanospheres or Nano-capsules	Polymer NPs possess excellent biocompatibility, biodegradability, and non-toxicity; thus, they are widely utilized in various applications such as drug delivery for tumors, wound dressings, and surgical scaffolds	Ashoka et al. (2023), Tian et al. (2022a), Xia et al. (2021), and Madej et al. (2022)
Inorganic NPs	Carbon-based NPs	Fullerenes and Carbon Nanotubes (CNTs)	Primarily used for delivering anti-cancer drugs, genes, and proteins for tumor chemotherapy	Lisik And Krokosz (2021) and Mahor et al. (2021)
	Metal NPs	Alkali metals and noble metals such as Cu (copper), Ag (silver), and Au (gold)	Noble metal-based nanoparticles are primarily applied in the medical field where high biocompatibility, stability, and avoidance of organic solvents are required for large-scale production	Roshani et al. (2023), Yaqoob et al. (2020), and Zhang et al. (2024)
	Ceramic NPs	Often found in amorphous, polycrystalline, dense, porous, or hollow forms	Commonly used in areas such as bone repair, photocatalysis, dye degradation through photolysis and imaging	Nizami et al. (2022), Kaushik et al. (2021)
	Semiconductor NPs	Possess characteristics that lie between metals and non-metals	Commonly used in photocatalysis, optical applications, and electronic devices	Okoro et al. (2023)

**FIGURE 1:**
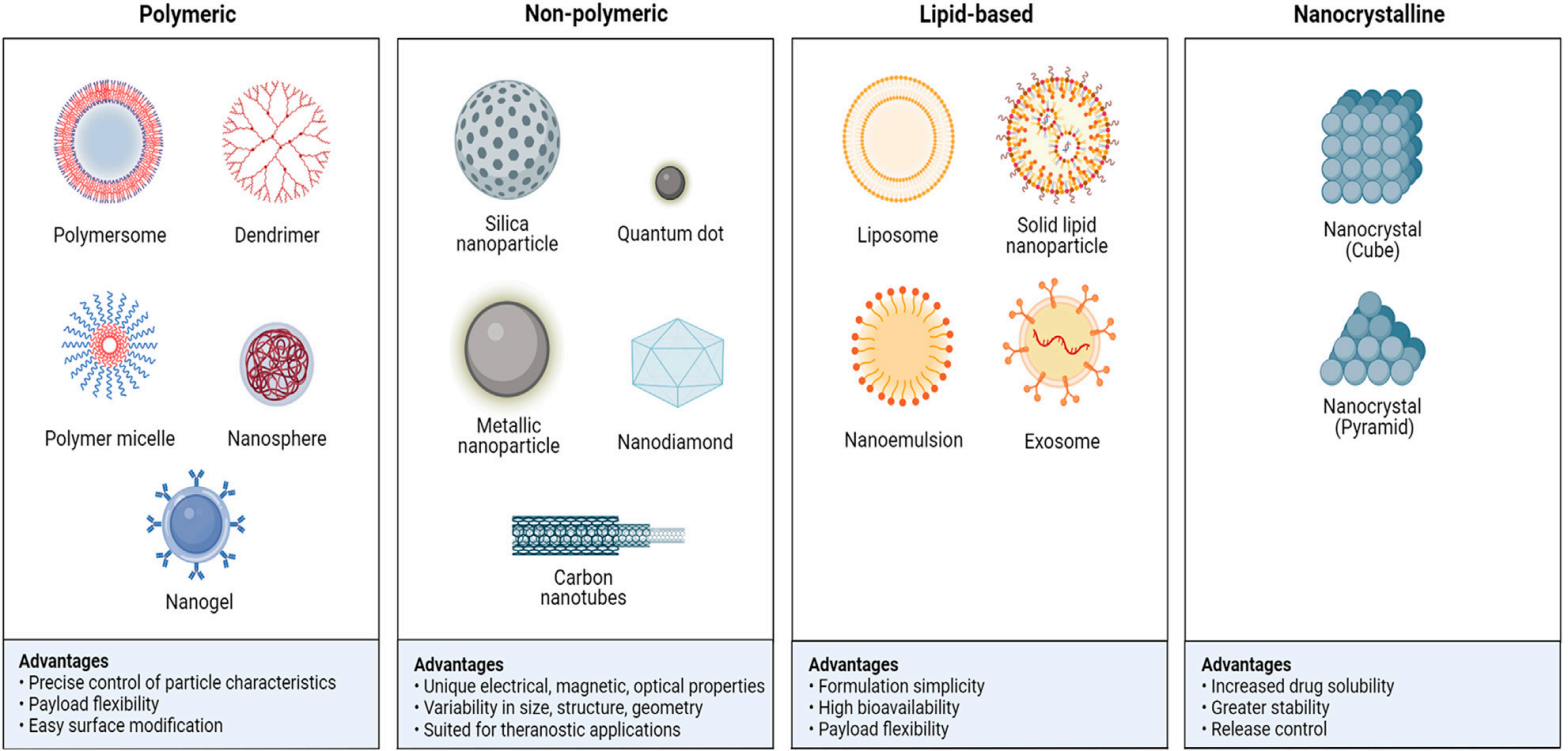
Each class of nanoparticle (NP) has numerous broad advantage. Adapted from Altemimi et al. (2024).

Traditional drugs have long been limited in clinical applications due to issues such as water solubility, stability, pharmacokinetics, low bioavailability, poor targeting, and toxicity (LI et al., 2023a). The emergence of nanomedicine has overcome these limitations and made significant strides (Altemimi et al., 2024; Zambonino et al., 2023). The application of nanomaterials in clinical diseases offers distinct advantages (Figure 1). Firstly, compared to most traditional drugs, nanomaterial formulations typically contain multiple functional components, each capable of exerting different effects on drug efficacy and safety throughout the treatment process (Zhou et al., 2021; Briolay et al., 2021; Mitchell et al., 2021). Secondly, some nanomaterials such as gold nanoparticles (AuNPs) possess unique electromagnetic properties, making them useful for photothermal therapy, photodynamic therapy, radiation therapy, X-ray imaging, computed tomography (CT), etc. (Fan et al., 2020; Batool et al., 2023). Thirdly, nanomaterials can distinguish pathological tissues from normal tissues, primarily based on the interactions between nanoparticles and biological tissues *in vivo* (Fan et al., 2020; Batool et al., 2023). Nanomedicine is an evolving field involving materials, devices, and technologies at the nanoscale, which can be used for the diagnosis, treatment, and prevention of clinical tumors (Elmowafy et al., 2023; Zheng et al., 2024). Current applications of nanoparticles include drug delivery and treatment such as nanoparticle Drug Delivery Systems (NDDS) (Ordikhani et al., 2021; Tu et al., 2023), diagnostics and imaging (Wang, 2020), tissue engineering and regenerative medicine (Wang et al., 2022), and immunotherapy (Li et al., 2023b). Additionally, the clinical application of nanomedicine is moving towards personalized medicine and integrated diagnosis and treatment to achieve more efficient clinical care, bringing about more innovations and breakthroughs in the field of medicine (Cui et al., 2024).

### 2.2 Personalized medicine for tumor

Personalized therapy, also known as individualized medicine or precision medicine, aims to reveal differences in individual responses, disease mechanisms, and risk factors during the disease process through high-throughput biomedical testing methods such as DNA sequencing, proteomics, imaging, and monitoring devices. This approach then determines how to optimize treatment, monitoring, or prevention of diseases to achieve the best clinical outcomes (Zhan et al., 2022; Desar and Rothermundt, 2023; Corrado and Garganese, 2022). It is widely recognized that there are certainly heterogeneities in the development of many diseases, so when diagnosing, treating, or monitoring specific patients, therapy must be tailored or personalized based on the unique biochemical, physiological, environmental exposures, and behavioral characteristics of individuals (Fujiwara et al., 2022).

Tumor or cancer is a dynamic disease, characterized by the transformation of non-malignant cells into malignant ones primarily through a series of pathological changes. This transformation leads to enhanced cell proliferation, evasion of growth suppression and cell death signals, induction of angiogenesis, and ultimately activation of programs that cause tissue invasion and metastasis (Brown, 2023; Mo et al., 2021). The occurrence and progression of tumors do not follow a fixed process but should be viewed as overall instability in key cellular processes. Even after completing malignant transformation, tumors continue to develop dynamically, undergoing continuous evolution. Eventually, they give rise to tumor masses composed of cancer cells with different molecular characteristics, exhibiting varying levels of sensitivity to cancer treatment (Zhang and Fu, 2021).

This heterogeneity may result in the uneven distribution of genetically distinct tumor cell subpopulations between and within disease sites (spatial heterogeneity), or temporal changes in the molecular composition of cancer cells (temporal heterogeneity) (Li et al., 2022). Therefore, accurately evaluating tumor heterogeneity and implementing personalized treatments targeting this heterogeneity are crucial in the clinical diagnosis and treatment of cancer. The development of molecular diagnostic techniques allows for a more accurate understanding of the genetic characteristics and molecular biomarkers of tumors, providing crucial information for devising personalized treatment plans (Guan et al., 2023). By analyzing the genome, transcriptome, and epigenetics information of a patients, clinicians can more accurately determine treatment strategies (Wu et al., 2019; Han et al., 2022). At the same time, immunotherapy and targeted therapy are important directions in the field of personalized cancer treatment. Immunotherapy harnesses the patient’s own immune system to attack cancer cells, while targeted therapy intervenes in the cancer process by targeting specific molecular targets (Letai et al., 2022). Personalized tumor treatment is a promising field that offers new possibilities for improving the treatment outcomes and survival rates of cancer patients. With advancements in technology and medicine, we can expect to see more personalized treatment options emerging, bringing hope to cancer patients. Currently, nanomedicine, as an emerging approach to tumor diagnosis and treatment, is mainly applied in personalized tumor treatment for targeted drug delivery, imaging diagnosis, and treatment monitoring (Shrestha et al., 2023; Alghamdi et al., 2022; Yang et al., 2020).

### 2.3 Correlation between nanomaterials with tumor personalized medicine

As an advanced approach to tumor diagnosis and treatment, nanomedicine combines nanomaterials with medical science, offering innovative methods for personalized cancer therapy. Currently, the application of nanomedicine in personalized cancer treatment is mainly focused on targeted drug delivery, imaging diagnosis, and treatment monitoring. In the area of targeted drug delivery, nanomaterials can be designed with specific biocompatibility and targeting capabilities, allowing for precise delivery to tumor tissues or cancer cells, thereby minimizing side effects on normal tissues (Shrestha et al., 2023; Alghamdi et al., 2022). For imaging diagnosis, nanomedicine provides efficient tools for early diagnosis and real-time tumor monitoring. Nanoprobes and nano-contrast agents, with their unique optical, magnetic, and radioactive properties, can significantly enhance imaging quality, making cancer cells more visible in early diagnostic stages (Yang et al., 2020). This precise imaging technology not only increases the likelihood of early cancer detection but also enables doctors to assess tumor size, location, and spread with greater accuracy, providing essential support for subsequent treatment decisions (Pallares et al., 2022). In treatment monitoring, nanotechnology also plays a crucial role. By designing smart nanomaterials, it is possible to monitor and provide real-time feedback on treatment effectiveness through drug release tracking or biomarkers produced during tumor response, which can help physicians adjust treatment plans in a timely manner, improving treatment efficacy and the level of personalization (Ju and Cho, 2023). Overall, the application of nanomedicine in personalized cancer treatment greatly expands the limitations of traditional therapies, making treatment more precise, effective, and personalized. With the continuous advancement of nanotechnology, the potential of nanomedicine in the field of oncology will further unfold, providing cancer patients with more optimized treatment options and extended survival. In the future, as the design and fabrication of nanomaterials improve, nanomedicine is expected to play a more critical role in precision oncology, becoming an indispensable part of cancer diagnosis and treatment.

## 3 Nanomaterials and tumor imaging diagnosis/monitoring

**FIGURE 2:**
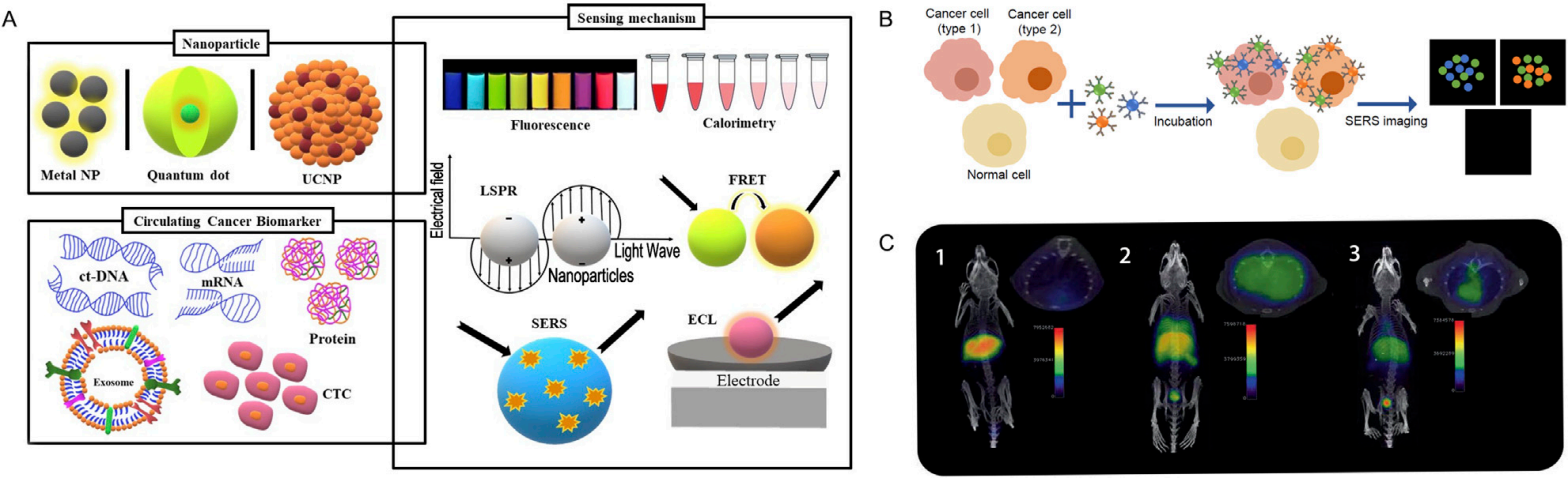
Nanomaterials and imaging diagnosis and monitoring. **(A)**. Schematic representation of the nanoparticles and the main sensing mechanisms of circulating cancer biomarkers. Adapted from Amri et al. (2021). **(B)**. Schematic illustration of simultaneous detection of three tumour associated antigens expressed different types of cancer cells of SERS imaging. Adapted from Jarockyte et al. (2020). **(C)**. (1) PET/CT imaging of a C57BL/6 mouse, without lipopolysaccharide (LPS) instillation, 1 h post i.v. injection with 68Ga-NRT-cFLFLF. (2) PET/CT imaging of a mouse with LPS-induced pulmonary inflammation 1 h post i.v. injection with 68Ga-NRT-cFLFLF. (3) PET/CT image of a neutrophil-depleted LPS-treated mouse 1 h post i.v. injection with 68Ga-NRT-cFLFLF. Adapted from Pellico et al. (2017).

Currently, The most widely used imaging modalities, including CT, MRI, X-ray, endoscopy, and ultrasound, can only identify tumors when they manifest as visible tissue changes, by which point many tumor cells may have multiplied or even reached distant locations (Houvast et al., 2020). Additionally, current imaging examinations still have limitations in the differential diagnosis of many benign and malignant lesions. Therefore, the development of early cancer detection technologies before tumor malignancy or metastasis is a major challenge. Despite the fact that nanotechnology has not yet been applied in clinical settings to diagnose cancer, it has already been marketed in various medical diagnostics and screenings, such as the application of gold NPs in early pregnancy testing (Bai et al., 2020). One important advantage of applying NPs to cancer detection is their relatively large surface area-to-volume ratio compared to bulk materials. Due to this characteristic, the surface of NPs can densely cover antibodies, small molecules, peptides, aptamers, and other substances, which can bind to and identify specific cancer molecules (Dai et al., 2021). Multivalent effects can be produced by delivering different binding ligands to cancer cells, increasing the detection’s sensitivity and specificity (Li et al., 2021). Real-time, practical, and affordable tools for cancer diagnosis and monitoring are being developed using nanotechnology-based diagnostic techniques. Research on the use of nanomaterials for tumor imaging diagnosis and monitoring mainly focuses on extracellular tumor biomarkers, tumor cells, and *in vivo* imaging (Figure 2).

### 3.1 Tumor biomarker detection

Measurable biomolecules that can be found in blood, other tissues, or bodily fluids like saliva and urine are known as tumor biomarkers. By determining their expression levels, it is possible to assess the presence of tumors in the body and their malignancy (Jardim et al., 2021). Biomarkers may include proteins, carbohydrates, or nucleic acids such as DNA, RNA, circRNA, mainly secreted by the organism or tumor cells (Deng et al., 2022). Assessing particular biomarker levels can assist track the effectiveness of treatment and help detect cancers or tumor recurrence early. However, biomarker detection is subject to certain limitations, including low biomarker concentrations in body fluids, variability in biomarker abundance within patients, and heterogeneity across different times and tissues (Pessoa et al., 2020). Nanotechnology offers the ability for high selectivity, sensitivity, and simultaneous measurement of multiple targets. Nanomaterials and nanoparticles can be used to improve biosensors and provide targeted imaging. Meanwhile, Because of the higher surface area-to-volume ratio that nanoparticles offer, biosensors are better able to detect the presence of particular biomolecules (Boehnke et al., 2022). Semiconductor NPs, metal NPs, and polymer NPs are three commonly used types of NPs probes for detecting tumor biomarkers (Combes et al., 2021; Amri et al., 2021) (Figure 2A).

Semiconductor NPs are commonly used for the detection of biomarkers due to their unique properties, including high quantum yield and molar extinction coefficient, broad absorption spectrum and narrow, efficient Stokes shift, high resistance to photobleaching, and excellent degradation resistance (Fatima et al., 2021). Li et al. (2011) proposed a novel approach for dual-protein immunoassay based on dual-color quantum dots. In this method, two lung cancer biomarkers, carcinoma-embryonic antigen (CEA) and neuron-specific enolase (NSE), were targeted (Li et al., 2011). CEA, one of the most common cancer biomarkers, has been utilized for monitoring cancer treatment efficacy and predicting tumor recurrence in late-stage cancer patients’ post-surgical resection (Hori et al., 2022). NSE is an enzyme that catalyzes the conversion of 2-phosphoglycerate to phosphoenolpyruvate, and it is associated with neuroendocrine cells, small cell lung carcinoma, and pancreatic islet cell tumors (Sandroni et al., 2022). After secretion, they can be detected at concentrations exceeding 15 ng/mL, with detection limits for each reaching 1.0 ng/mL. The study results indicate that CEA and NSE can be sensitively detected using a conventional 96-well fluorescence plate reader, with detection limits of 1.0 ng/mL for each target. Within the calibrated range, this approach demonstrates excellent precision for each target, with an accuracy of around 0.53%. Furthermore, the method has been successfully applied to the determination of dual biomarkers in actual samples, showing no cross-reactivity. It also exhibited good correlation when compared with traditional assays for CEA and NSE in human serum samples from 25 individuals (Hu et al., 2023). Additionally, research has shown the development of a precise enzyme-induced targeting gold nanoparticle system mediated by iRGD/AuNPs-A&C, which utilizes tumor-targeting penetrating peptide iRGD modified gold NPs. This system exhibits high permeability and aggregation in breast tumor cells (Liang et al., 2023). Tong et al. (2010) prepared Cy5-conjugated poly (lactic acid) (Cy5-PLA) nanoparticles through nanoprecipitation. They conjugated Cy5-PLA NPs with oligonucleotides (aptamers) binding to prostate-specific membrane antigen (PSMA). Aptamers possess high affinity and strong binding specificity to their respective targets, including ions, bacteria, peptides, viruses, lipids, and even whole cells. Polymer NPs (Cy5) bound by A10 RNA aptamer can bind to PSMA. Cy5-PLA/aptamer NPs can only bind to cells that respond positively to PSMA, such as LNCaP and canine prostate cancer cells; they are unable to bind to PC3 cells, which respond negatively to PSMA (Tong et al., 2010).

### 3.2 Tumor cell detection

Prior to invading the circulatory microvasculature and lymphatic system, the primary tumor initially invades the surrounding tissues. Subsequently, they survive in the bloodstream and migrate to distant tissues’ microvasculature, ultimately extravasating and surviving in the microenvironment of distant tissues. This provides an appropriate heterogeneous microenvironment for the development of macroscopic secondary tumors (Xiao and Yu, 2021). Accurately identifying circulating tumor cells (CTCs), or metastatic cancer cells in the bloodstream, may influence the prognosis and diagnosis of cancer (Lin et al., 2021). CTCs have been extensively researched as a component of liquid biopsy because of their potential uses. Compared to tumor biomarker detection, CTCs detection has the advantage of directly and specifically identifying and isolating tumor cells in the bloodstream, providing direct information on metastasis risk and cancer progression. It is widely used for early detection and dynamic monitoring of metastatic cancers, offering significant advantages in metastasis risk assessment, prognosis analysis, and personalized treatment evaluation. However, its clinical application is limited by the complexity and high cost of the detection process, as well as the need for specialized equipment and technical expertise.

Nanomaterials offer significant advantages for CTCs detection as they can efficiently adsorb targeting ligands with the ability to recognize specific molecules on cancer cells (Chen et al., 2019; Jarockyte et al., 2020; Choi et al., 2010). Thus, the isolation of CTCs has good specificity and recovery rates, improving the sensitivity of detection. Research has reported various types of nanomaterials, such as metal NPs, semiconductor NPs, carbon nanotube NPs, and polymer NPs for CTCs detection, showing potential to advance cancer diagnosis and prognosis (Jarockyte et al., 2020; Wu et al., 2022) (Figure 2B). Zhao et al. (2024) constructed a surface with nanoscale roughness by coating a cellulose acetate membrane with nanoparticles formed by the polymerization of melamine and furfural, enabling the efficient capture of sparse CTCs in blood samples. Subsequently, the CTCs on the surface can be quantitatively detected by colorimetry with the aid of a COF-based nanozyme, achieving a detection limit (LOD) as low as 3 cells/mL (Zhao et al., 2024). Meanwhile, Lu et al. (2023) constructed a chip substrate by crosslinking silica nanoparticles layer-by-layer on glass slides using polyacrylic acid. Polyacrylic acid was immobilized as a spacer, with capture ligands attached to it. The results demonstrated that this chip could be integrally applied for CTC capture, post-treatment, and imaging detection. In samples with 9 cells/mL and clinical blood samples (7.5 mL), the detected cell counts were 33 and 40, respectively, with a detection rate of 100% for positive samples (Lu et al., 2023). Overall, nanomaterials efficiently capture and enrich rare CTCs through their surface properties; they improve detection sensitivity by combining with specific molecules to recognize tumor biomarkers; and they can enhance imaging signals, thereby increasing detection accuracy. Nanomaterials significantly enhance the sensitivity, specificity, and operability of tumor cell detection, advancing the development of early tumor diagnosis and personalized treatment.

### 3.3 *In vivo* imaging

Apart from the *ex vivo* diagnosis of cancer through the identification of cancer cells and biomarkers in liquid biopsy samples, there are many advantages to identifying cancer tissue *in vivo* for cancer diagnosis and treatment. The continuous advancement of technology has improved diagnostic imaging techniques, enabling the detection and diagnosis of early-stage diseases (Le Fur et al., 2021). Although various imaging modalities such as magnetic resonance imaging (MRI), computed tomography (CT), positron emission tomography (PET), and single-photon emission computed tomography (SPECT) are widely used in clinical practice, each imaging technique has its advantages and limitations. Currently, the development of integrated PET/MRI scanners has opened new prospects for cancer diagnosis, treatment, and follow-up. In recent years, radionuclide imaging, fluorescence imaging, magnetic resonance imaging, ultrasound imaging, photoacoustic imaging, and multimodal imaging are all examples of nanoparticle-based tumor molecular imaging methods (Pellico et al., 2017) (Figure 2C).

Molecular imaging uses multimodal imaging to obtain information at the biological and cellular levels, thereby tracking biological pathways and discovering many typical tumor characteristics. In this context, contrast agents (CAs) based on nanoparticles (NPs) can improve the biocompatibility and biodistribution of probes, extend the blood half-life to achieve accumulation at specific targets, and ensure non-toxicity to surrounding tissues (Milan et al., 2022; Fernández-Barahona et al., 2018; Ko et al., 2022; Yin et al., 2024). Additionally, CAs can deliver drugs or conventional therapeutic agents simultaneously, achieving dual diagnostic and therapeutic effects, thereby advancing the process of personalized treatment. NPs have been widely studied in preclinical stages as imaging probes for dual MRI/PET tumor imaging, with the most common carriers being iron oxide NPs and silica NPs (Karageorgou et al., 2023; Zhao et al., 2023).

The capacity of magnetic iron oxide nanoparticles (NPs), which are usually made of magnetite Fe_3_O_4_ and maghemite γ-Fe_2_O_3_, to shorten T2 and T2* relaxation durations makes them useful for MRI imaging, especially in the liver, spleen, and bone marrow. They can be divided into four categories based on size: monocrystalline iron oxide (MION), ultrasmall superparamagnetic iron oxide (USPIO), superparamagnetic iron oxide (SPIO), and micrometer-sized paramagnetic iron oxide (MPIO) (Qiao et al., 2023). Xie et al. (2010) tagged human serum albumin matrices with Cy5.5 dye and ^64^Cu-DOTA after encasing dopamine-modified Fe3O4 NPs. Injection of these nanoparticles into U87MG xenograft mouse models revealed higher signal-to-noise ratios in PET and NIRF imaging compared to MRI, indicating higher sensitivity. Conversely, MRI scans after nanoparticle injection exhibited clear but uneven distribution due to their high spatial resolution (Xie et al., 2010). Lee et al. (2008) conjugated RGD with ^64^Cu-labeled Fe_3_O_4_ nanoparticles and conducted imaging on U87MG mouse models, where both PET and MRI confirmed nanoparticle accumulation mediated by αvβ3 integrin binding. Kim et al. (2013) injected ^68^Ga-labeled Fe_3_O_4_ NPs into colorectal cancer nude mice and found suppression of cancer cell proliferation, induction of apoptosis, and cancer cell death. PET/MRI scans yielded detailed pictures and accurate tumor area measurement (Kim et al., 2013). In addition, iron nanoparticles can serve as an adjunct in clinical imaging diagnosis to help differentiate between benign and malignant tumors. A prospective study in pediatric cancer patients found that the iron supplement Ferumoxytol can enhance image contrast in MRI scans, aiding in the clearer visualization of tissue and lesion details, and assisting in distinguishing between benign and malignant lymph nodes in children with cancer (Muehe et al., 2020).

Silicon dioxide-based NPs are considered ideal biocompatible matrices that can be integrated into imaging probes, with two main categories: solid (SiNPs) and mesoporous (MSNs). MSNs are usually used for molecular, multimodal, CT, MRI, and PET imaging, whereas SiNPs are commonly used as optical imaging agents. MSNs are created using sol-gel techniques with surfactant templates, possessing high surface areas, tunable sizes, shapes, and pore structures, as well as appealing features easily functionalized through synthetic approaches (Shuai et al., 2023). Imaging of sentinel lymph nodes (SLNs), as the first line of defense against primary tumor metastasis, is regarded as a crucial strategy clinically for tracking tumor metastasis. Using various conjugation strategies, Huang et al. integrated three imaging probes—the positron emission radiotracer ^64^Cu, the T1 contrast agent Gd-DTTA, and the near-infrared (NIR) dye ZW800—with PET and MRI imaging probes dispersed on the surface and inside the mesoporous channels of NPs. Compared to normal SLNs, multifunctional MSNs probes exhibited faster uptake rates and higher uptake ratios in T-SLNs, confirming the feasibility of these MSNs probes as contrast agents for mapping SLNs and identifying tumor metastasis (Huang et al., 2012) (Figure 2C).

Additionally, ultrasound has long been used in clinical practice for cancer detection and image-guided tissue biopsy. For decades, stable perfluorocarbon microbubbles have been used as contrast agents in ultrasound imaging. However, the large size, poor stability, and short cavitation activity of microbubbles have limited their application in cancer imaging and therapy (Sabuncu and Yildirim, 2021). Nanoparticles is expected to address this issue. Research by Pucci et al. found that piezoelectric hybrid lipid-polymer nanoparticles can effectively encapsulate non-genotoxic drugs (nutlin-3a) and be functionalized with peptides (ApoE) to enhance their passage through the blood-brain barrier. Under ultrasound stimulation, the nanocarrier can reduce cell migration, actin polymerization, and glioma cell invasiveness while promoting apoptotic and necrotic events (Pucci et al., 2022). Zhang et al. (2023) developed a novel nanoparticle with a PLGA outer layer, loaded with perfluoropentane (PFP), paclitaxel (PTX), and an anti-miR-221 inhibitor, naming it PANP. RAW-PANPs successfully stopped the growth and progression of tumors *in vivo* as well as the proliferation of TNBC cells *in vitro*. These therapies did not harm the kidneys, liver, or heart. In addition to creating a chemotherapeutic system that targets cancer cells and is delivered by macrophages and activated by ultrasound, their research also showed promise for clinical use by demonstrating a miRNA-based method to increase drug sensitivity in cancer cells (Zhang et al., 2023).

## 4 Nanomaterials and targeted drug delivery

Nanoparticle drug delivery systems (NDDS) are drug delivery systems that integrate drugs into various nanocarriers, allowing for drug delivery concentrated in targeted tissues/organs. NDDS exhibits high drug stability, potential for sustained release, and low drug toxicity (Li et al., 2020; Du et al., 2024). By increasing drug concentrations in the bloodstream, NDDS can prolong the half-life of drugs and improve the solubility, stability, and bioavailability of hydrophobic drugs, thereby reducing the frequency of drug delivery (Zou et al., 2021). According to the action mechanisms, NDDS can be classified as Passive Targeting Drug Delivery Systems (PTDDS), Active Targeting Drug Delivery Systems (ATDDS), and Environmental Responsive Targeting Drug Delivery Systems (ERTDDS) (Tian et al., 2022b; Hristova-Panusheva et al., 2024; Kebebe et al., 2018; Majumder and Minko, 2021; Mi, 2020), see Table 2 for details. NDDS has been widely researched and applied in the clinical treatment of various tumors.

**TABLE 2:** Advantages and disadvantages of NDDS and their applications in tumors.

Classification	Advantages	Disadvantages	Applications	References
PTDDS	Simple and easy No need for complex modification Low cost More applicability and safety	Low targeting efficiency Susceptible to individual patient differences Limited efficacy	Liposomal Adriamycin and daunorubicin	Ji et al. (2024)
ATDDS	High specific targeting Reduce side effects and improve curative effect High delivery efficiency and retention	Complex modification process High difficulty and cost; may trigger immune response Various in different patients	Experimental studies and early clinical trials	Lv et al. (2018)
ERTDDS	Responsive and self-regulating Precise control of drug release; combined with PTDDS or ATDDS	Strongly dependent on microenvironmental conditions complex carrier system structure High preparation process Not stable as traditional system	Experimental studies	Chen et al. (2023b)

### 4.1 PTDDS

**FIGURE 3:**
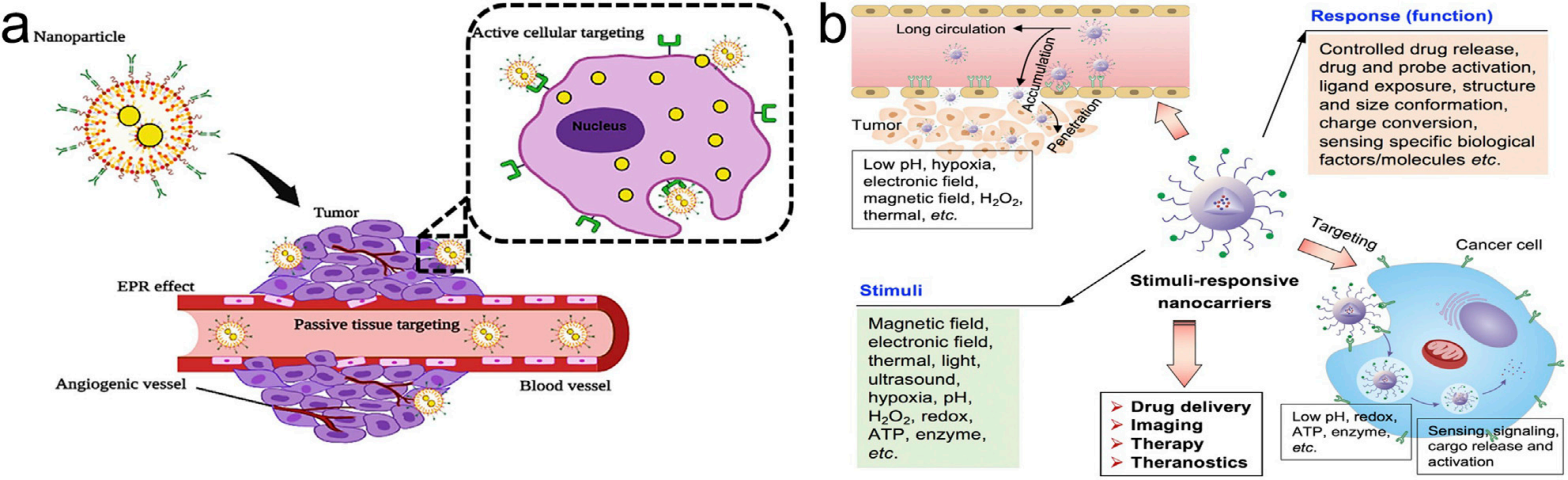
Nanoparticle drug delivery systems (NDDS). **(A)** Passive Targeting Drug Delivery Systems (PTDDS) and Active Targeting Drug Delivery Systems (ATDDS). Adapted from Batool et al. (2023); **(B)** Environmental Responsive Targeting Drug Delivery Systems (ERTDDS). Adapted from Mi (2020).

PTDDS is primarily based on the enhanced permeability and retention (EPR) effect in tumor tissues, delivering drugs to tumor tissues rather than normal tissues. Drug-loaded NPs, such as liposomes and polymer NPs, are naturally engulfed through physiological processes like cellular uptake to achieve targeted drug distribution (Maeda et al., 2000). Organic NPs, including liposomes and polymer NPs, are widely used in PTDDS (Batool et al., 2023; Tian et al., 2022b) (Figure 3A).

#### 4.1.1 Lipid NPs, LNPs

LNPs are among the first nanoparticles studied as drug carriers. They consist of phospholipid molecules with hydrophilic heads and hydrophobic tails, which self-assemble into bilayer spherical vesicles. Drugs are either encapsulated within the hydrophilic core or embedded in the lipid membrane of LNPs, allowing them to enter tumor tissues through the bloodstream (Van Kammen et al., 2023; Wang et al., 2024). Gao et al. (2015) aimed to enhance the therapeutic efficiency of temozolomide (TMZ) for glioblastoma treatment by preparing TMZ-loaded LNPs (TMZ-LNPs) using the precursor liposome method. The average size of TMZ-LNPs was (156.70 ± 11.40) nanometers, with mean drug encapsulation efficiency and drug loading capacity of (35.45 ± 1.48)% and (2.81 ± 0.20)%, respectively, at a pH of 6.46. *In vitro* release studies conducted via dynamic dialysis revealed that TMZ released slowly from LNP compared to the solution group, with TMZ-LNP having a 3.57-fold increase in t (1/2β) and a 1.27-fold increase in mean residence time (MRT) compared to TMZ solution. The C_max_ and AUC values of TMZ-LNPs were 1.10-fold and 1.55-fold higher than those of TMZ solution, respectively. Additionally, biodistribution studies in mice indicated that after intravenous injection, TMZ-LNPs reduced accumulation in the heart and lungs while increasing drug concentration in the brain, suggesting that TMZ-LNPs enhanced intracranial therapeutic efficacy and reduced pulmonary and cardiac toxicity (Gao et al., 2015). Additionally, researchers have discovered a self-assembling α-melittin-LNPs system that carry without additional tumor antigens but promotes the release of endogenous tumor antigens and enhances the activation of antigen-presenting cells (APCs) in lymph nodes. *In vivo*, α-melittin-LNPs significantly inhibits the growth of primary and distant tumors, with suppression rates reaching 95% and 92%, respectively (Yu et al., 2020). Resistance to immune checkpoint inhibitors (ICIs) is a major hurdle in cancer immunotherapy. LNPs containing stimulator of interferon genes (STING) agonists, referred to as STING-LNPs, effectively induce anti-tumor activity by activating NK cells. This results in increased expression of CD3, CD4, NK1.1, PD-1, and IFN-γ in tumor tissues of melanoma lung metastasis mice. Consequently, the expression of programmed cell death ligand 1 (PD-L1) in tumor cells is enhanced, leading to a synergistic anti-tumor effect when combined with anti-PD-1 therapy (Nakamura et al., 2021).

With the approval of mRNA vaccines for the novel coronavirus, mRNA therapy has become a hot topic in the biomedical field. Simultaneously, mRNA has shown immense potential in cancer therapy. Consequently, targeted delivery of mRNA encapsulated in LNPs has become a current research hotspot (Zong et al., 2023). Various cancer vaccines based on LNPs-mRNA are currently undergoing clinical trials. Sahin et al. (2020) designed a mRNA vaccine named FixVac (BNT111) targeting four commonly expressed non-mutated tumor-associated antigens in melanoma. This vaccine was administered intravenously to patients with advanced melanoma after encapsulation in LNPs. In a Phase I clinical trial (NCT02410733), it was found that after the sixth immunization, metabolic activity in the spleen increased. After the eighth immunization, over 75% of patients developed immune responses against at least one tumor-associated antigen (Sahin et al., 2020). Meanwhile, new antigens generated by cancer cell mutations are typically different among different cancer patients, making the development of personalized vaccines an urgent issue. mRNA-4157 is a personalized cancer vaccine encoding 34 new antigens, and its Phase I clinical trial (NCT03897881) has evaluated the immunogenicity of mRNA-4157 alone and in combination with immune checkpoint inhibitors for patients with respectable and unresectable solid tumors. In the monotherapy group, 14 out of 16 patients maintained disease-free status during the study period, with a median progression-free survival of 8 months. The overall response rate in the combination group was 50%, with a median progression-free survival of 9.8 months (Burris et al., 2019). Using a similar LNPs formulation, mRNA vaccines have also been shown to induce specific T cell responses in patients with gastrointestinal cancers (NCT03480152) (Cafri et al., 2020). Additionally, researchers have found that LNPs can be used to deliver mRNA encoding CLDN6, a target for CAR-T cell therapy in solid tumors. Intravenous injection of the vaccine promotes CLDN6 expression on dendritic cells and macrophages in the spleen, activates CLDN6-CAR-T cells, and inhibits the growth of mouse solid tumors (Reinhard et al., 2020).

#### 4.1.2 Polymer NPs

Polymer NPs are solid colloidal systems in which drugs can be encapsulated, dissolved, adsorbed, or encapsulated into the polymer matrix (You et al., 2024). The structure of these NPs may vary depending on the preparation method. This variation may range from nano-capsules (drug confined within vesicles surrounded by a single polymer membrane forming an aqueous or oily cavity reservoir system) to nanospheres (drug dispersed throughout the entire particle matrix) (Su et al., 2020). Several polymers have been developed for passive targeting delivery of drugs, such as polylactic acid (PLA), chitosan, polyacetal acid, polycaprolactone (PCL), polyethylene glycol (PEG), and poly (lactic-co-glycolic acid) (PLGA) (Song et al., 2022). There are some researchers have found that NPs loaded with paclitaxel (PTX) based on PEG-PLGA display more than three times the cytotoxicity on HeLa cells compared to standalone paclitaxel solution. This increased activity is attributed to the enhanced permeability and retention (EPR) effect induced by paclitaxel-loaded NPs (Danhier et al., 2009). Meanwhile, cancer therapy is often hindered by multidrug resistance (MDR), which reduces the chemotherapy activity of drugs and can even lead to treatment failure. Therefore, an effective carrier is needed to simultaneously deliver multiple drugs into tumors. Zou et al. (2017) co-loaded paclitaxel (PTX) and the P-glycoprotein (P-gp) inhibitor borneol (BNL) into PEG-PAMAM NPs using a one-step nanoprecipitation method. These NPs exhibited high drug loading efficiency, narrow size distribution, and low hemolysis rate. Drug efflux testing and molecular docking models confirmed that the combination of PTX and BNL increased the intracellular concentration of PTX in A2780/PTX cells. Moreover, compared to free PTX and PTX + BNL, PB/NPs (PEG-PAMAM nanoparticles) and P/NPs with added BNL (PTX nanoparticles and free BNL) demonstrated higher cellular uptake and cytotoxicity in A2780/PTX cells, while also reducing mitochondrial membrane potential and enhancing apoptosis (Zou et al., 2017).

### 4.2 ATDDS

Compared to the low selectivity and affinity of PTDDS, ATDDS utilize various targeting molecules such as proteins, antibodies, small molecules, or nucleic acids to modify drug-loaded NPs. These modifications enable the directed delivery of drugs to tumor tissues through targeted interactions (Tian et al., 2022b). The main types of ATDDS include receptor-mediated NPs, peptide-mediated NPs, and small molecule-mediated NPs (Batool et al., 2023; Mi, 2020) (Figure 3A).

#### 4.2.1 Receptor-mediated NPs

In most tumors, especially rapidly growing malignant cancers, overexpression of receptors is common and often associated with poor disease prognosis. Receptor-mediated endocytosis is one of the main mechanisms of action for anti-tumor drugs. Researchers modify nanocarriers with corresponding proteins to enhance the targeting of drugs. Charbgoo et al. (2018) covalently synthesized single-walled carbon nanotube (SWNT) NPs conjugated with folate receptors and found that SWNTs larger than 450 nm are more suitable for targeting tumor cells via receptor-mediated pathways (Charbgoo et al., 2018). Gabizon et al. (2010) investigated and found that PEG-doxorubicin LNPs conjugated with folate demonstrated superior activity compared to free doxorubicin, both in human nasopharyngeal carcinoma cell lines and in tumor models induced by cisplatin-resistant derivatives (Gabizon et al., 2010). DI17E6 is a monoclonal antibody targeting αv integrin, which inhibits the growth of melanoma both *in vitro* and *in vivo*. By covalently binding DI17E6 to human serum albumin nanoparticles, specific targeting of αvβ3 integrin-positive melanoma cells is achieved. DI17E6 nanoparticles loaded with the drug doxorubicin exhibit higher cytotoxic activity in αvβ3-positive melanoma cells compared to free drug (Wagner et al., 2010). The transferrin receptor system is highly expressed on the surface of brain glioma stem cells and brain capillary endothelial cells, which means that targeting it opens up a channel for therapeutic molecules to reach the brain. Sun et al. (2017) targeted the subset of brain glioma stem cells, binding to transferrin and encapsulating cisplatin in NPs to evaluate their effectiveness in destroying tumor cells. The study found that the NPs were highly effective in penetrating the blood-brain barrier and delivering high doses of cisplatin, enhancing the cytotoxicity against glioma stem cells (Sun et al., 2017).

#### 4.2.2 Peptide-mediated NPs

Small molecules called peptides are made up of two to fifty amino acids. The linked peptides usually enter cells by receptor-mediated endocytosis after ligand peptides attach to receptors. This includes circulating RGD peptides (cRGD), internalizing RGD peptides (iRGD), cell-penetrating peptides (CPPs), etc. A study for the first time reported the modification of TAT peptide onto mesoporous silica NPs (MSNs) (designated as MSNs-TAT), achieving high payload nuclear-targeted drug delivery. It was found that MSNs-TAT with diameters of 50 nm or smaller effectively targeted the cell nucleus, facilitating targeted release of the active anticancer drug DOX into the nucleus, significantly enhancing the killing efficiency against cancer cells (Pan et al., 2012). Gu et al. (2013) used MT1-AF7p peptide to modify polyethylene glycol-polylactic acid (PEG-PLA) NPs loaded with paclitaxel (PTX) (designated as MT1-NP-PTX) and found significant anti-proliferative activity against glioma cells. Its IC50 decreased by 2.81-fold and 2.47-fold compared to free PTX and NP-PTX, respectively. Compared to PTX and NP-PTX, MT1-NP-PTX exhibited the highest percentage of apoptosis (Gu et al., 2013). And a type of gold NPs conjugated with a cell-penetrating peptide and cationic polyethyleneimine (AuPT) can compact pDNA into cationic nanocomplexes and penetrate intact stratum corneum, deeply reaching melanoma tissues without the need for additional enhancement techniques. Simultaneously, AuPT demonstrates extremely high efficiency in stimulating intracellular uptake of pDNA and nuclear targeting, holding immense potential for localized gene therapy (QI et al., 2023).

#### 4.2.3 Small molecule-mediated NPs

Small molecules such as folic acid (FA), biotin, and bisphosphonates are commonly used as ligands for targeted drug delivery systems in ATDDS, as their good safety profiles, lack of immunogenicity, and ease of modification. Folate receptors (FR) are highly expressed in many cancers but not in normal tissues, making folate the most widely used small molecule ligand. Research based on folate-modified PAMAM G5 dendrimer polymer nano-targeting systems for brain glioma DOX has shown a significant improvement in the treatment of gliomas. FA-PAMAM/DOX exhibited a tumor growth inhibition rate of 57.44%, whereas free DOX was only 17.70%. Additionally, compared to DOX alone, FA-PAMAM/DOX significantly reduced tumor volume in mice. Furthermore, the data showed that FA-PAMAM/DOX significantly prolonged the half-life and increased the accumulation of DOX in brain tumors, leading to an improvement in the median survival time of nude mice with xenograft tumors (Xu et al., 2016). Meanwhile, Jang et al. combined hypoxia-responsive glycol chitosan nanoparticles with FA (HRGF). *In vitro* drug release studies showed that doxorubicin (DOX) loaded HRGF (D^@^HRGF) NPs released faster under hypoxic conditions than under nonmonic conditions. Compared to D^@^HRG and free DOX, D^@^HRGF NPs demonstrated more effective anti-tumor activity in mice (Jang et al., 2020). Additionally, Alexandria et al. found that functionalized FA covalently bonded poly (styrene-alt-maleic anhydride)-SMA formed FA-DABA-SMA copolymer NPs through a biolinker, 2,4-diaminobutyric acid (DABA). Empty FA-DABA-SMA reduced tumor spheroid volume by interfering with FR signaling pathways by reducing HES1 and NOTCH1 protein expression levels. Moreover, FA-DABA-SMA induced apoptosis, further altering the morphology of tumor cells, and significantly reducing their migration capability (Decarlo et al., 2021).

### 4.3 ERTDDS

ERTDDS refers to the targeted distribution of drug-loaded NPs in the body through physical and chemical effects such as magnetic, thermal, acoustic, optical, electrical, and pH effects (Mi, 2020; Hu et al., 2022; Zhou et al., 2024b) (Figure 3B). Traditional drug delivery systems are not ideal as they fail to maintain stability at the target site and promote drug release. Over time, ERTDDS design has emerged as a research hotspot. ERTDDS can specifically react to endogenous or exogenous stimuli, such as physical properties (like photosensitivity), chemical properties (like pH sensitivity and reduction sensitivity), and biological properties (like matrix metalloproteinases), in order to comprehend the physiological differences between tumors and normal tissues (He et al., 2020; Cong et al., 2022). Only in proximity to the site of drug action can ERTDDS react to internal and external environmental stimuli, releasing medicines in tumor tissue while preserving stability in other tissues. As a result, it lessens toxicity and adverse effects while enhancing the anti-tumor activity.

Photodynamic therapy (PDT) and photothermal therapy (PTT) are the foundations of light-sensitive TDDS, a tumor treatment technique. After NPs reach the tumor site under light conditions, photosensitizers or photothermal agents absorb light and release reactive oxygen species, singlet oxygen, or induce local high temperatures (Guo et al., 2023). This selectively and efficiently generates cytotoxic activity, killing tumor cells while protecting normal tissues. Xu et al. (2018a) prepared silk fibroin NPs (SFNPs) loaded with the dye indocyanine green (ICG) to construct a therapeutic nanoplatform (ICG-SFNPs) for photothermal therapy of glioblastoma. *In vitro* studies demonstrated the slow release of ICG from ICG-SFNPs, with only (24.51 ± 2.27)% of encapsulated ICG released even after 72 h. Moreover, compared to free ICG, ICG-SFNPs exhibited a more stable photothermal effect upon exposure to near-infrared radiation. Local near-infrared irradiation rapidly increased the temperature at the tumor site, leading to tumor cell death. After treatment, tumor growth was completely suppressed, with a relative tumor volume of (0.55 ± 0.33), whereas free ICG exhibited a volume of (33.72 ± 1.90) (Xu et al., 2018a). Human blood and interstitial fluid normally have an alkaline pH of 7.4, whereas the tumor microenvironment has an acidic pH of about 5.6. This slight acidic environment is primarily due to the rapid proliferation of tumor cells and excessive accumulation of lactate (Johung and Monje, 2017). Therefore, numerous studies have utilized the pH difference between tumor tissue and normal tissue to construct drug delivery systems based on pH-responsive materials. During the transition from a weakly alkaline to a slightly acidic environment, many delivery methods may modify their physicochemical features, such as swelling and enhanced solubility. These further activate the release of packed drug molecules, which serve a targeted role in tumor therapy (Xu et al., 2018b). Li et al. (2012) synthesized a pH-sensitive dual-targeted drug carrier (G4-DOX-PEG-Tf-TAM) composed of PAMAM dendrimers conjugated with transferrin (Tf) and tamoxifen (TAM), aiming to enhance blood-brain barrier transport and increase drug accumulation in glioma cells. Their research showed that at pH 4.5, the release rate of DOX was 32%, while at pH 7.4, it was 6%, indicating relatively rapid drug release under weakly acidic conditions and stability in normal physiological environments (Li et al., 2012). Due to various reasons, including overexpression of drug efflux pumps like P-gp and inherent resistance caused by cancer stem cells (CSCs), breast cancer cells can develop resistance to treatment. Disulfiram (DSF) serves as an inhibitor of P-gp and CSCs. Swetha et al. developed tumor extracellular pH-responsive nanoparticles using PLGA conjugated with coenzyme Q10 polyol (TPGS) surface-modified with histidine ([DD]NpH-T). *In vitro* studies demonstrated that [DD]NpH-T exhibited increased drug release at pH 6.8, enabling better penetration of 3D tumor spheroids, reducing serum protein adsorption, and enhancing cytotoxicity against docetaxel-resistant breast cancer cells. *In vivo* studies showed significantly increased plasma area under the curve and tumor drug delivery with [DD]NpH-T, thereby improving the *in situ* anti-tumor effect in breast cancer, enhancing ROS expression levels and cell apoptosis, while reducing P-gp expression and preventing lung metastasis (Swetha et al., 2023).

## 5 Conclusion and perspectives

Personalized therapy for tumors involves treatment plans tailored to individual patient characteristics and disease traits. It is based on individual pathological and physiological features, genomics, genetics, and molecular characteristics of tumors, aiming to improve treatment efficacy and reduce adverse reactions. Currently, the focus of personalized therapy in oncology remains on genomics and molecular diagnostics, targeted therapy, immunotherapy, liquid biopsies, and circulating tumor DNA monitoring, among others. The emergence of nanotechnology offers promising directions for personalized tumor therapy, including drug targeting delivery systems, detection of biomarkers, *in vivo* imaging, and strategies for simultaneous diagnosis and treatment. Despite demonstrating significant potential in cancer treatment by providing unique molecular properties that enable more targeted and less toxic therapeutic approaches, overcoming certain obstacles is necessary to fully harness the advantages of nanotechnology in cancer therapy. Moreover, as the biocompatibility and metabolic pathways of nanomaterials are not yet fully understood. And certain metal nanoparticles (such as silver, gold, or copper nanoparticles) may exhibit toxic effects on organs such as the liver, kidneys, lungs, and nervous system. Concerns about the toxicity and safety of nanomedicine, including its effects on organs and the reproductive system, are crucial for long-term clinical applications. Additionally, The inability to find suitable animal models that closely mimic human tumor illnesses makes it difficult to convert preclinical research findings into clinical trials. To fully realize the potential of nanotechnology in transforming tumor therapy and delivering individualized and efficient cancer therapies, these obstacles must be overcome via creative thinking and thorough study.

In summary, nanomedicine holds the potential to overcome the limitations of traditional cancer treatments and has the prospect of fundamentally altering the approach to cancer therapy. Utilizing NPs-mediated drug delivery enables more precise tumor targeting and enhances drug efficacy across various treatments. The use of nanomedicine in cancer therapy has a bright future, despite obstacles include enhancing biocompatibility, controlling drug release, streamlining nanoparticle design, and creating suitable evaluation models. Sustained innovation will enable it reach its full potential and open the door to tumor treatment that is tailored to each patient.
